# A Lightweight Machine Learning Framework for Post-Stroke Gait Abnormality Classification Using Wearable Gyroscope Features

**DOI:** 10.3390/s26103143

**Published:** 2026-05-15

**Authors:** Stamatios Orfanos, Thanita Sanghan, Andreas Menychtas, Christos Panagopoulos, Ilias Maglogiannis, Surapong Chatpun

**Affiliations:** 1 Bioassist SA, 26504 Rio, Greece; sorfanos@unipi.gr (S.O.); cpan@bioassist.gr (C.P.); 2 Department of Biomedical Sciences and Biomedical Engineering, Faculty of Medicine, Prince of Songkla University, Songkhla 90110, Thailand; thanita.sa@psu.ac.th; 3 Department of Digital Systems, Faculty of Information and Communication Technologies, University of Piraeus, 18534 Piraeus, Greece; amenychtas@unipi.gr (A.M.); imaglo@unipi.gr (I.M.)

**Keywords:** gait classification, gyroscope features, machine learning, stroke rehabilitation, wearable sensors

## Abstract

**Highlights:**

**What are the main findings?**

- The random forest model achieved the highest F1-score (0.94) and near-perfect classification in the confusion matrix.
- Random forest and XGBoost showed excellent generalization, with high AUC values of 0.98 and 0.97 and minimal misclassification.

**What are the implications of the main findings?**

- Early screening and gait abnormalities detection.
- Objective rehabilitation outcome evaluations.

**Abstract:**

Accurately classifying gait abnormalities is crucial for the effective monitoring and rehabilitation of stroke patients. This study proposed a lightweight machine learning framework for distinguishing healthy from abnormal gait patterns using statistical features extracted from wearable gyroscope data. Statistical *z*-axis angular velocity values from both limbs were derived and used to evaluate the performance of multiple classifiers, including logistic regression, support vector machines, and ensemble methods. A leave-one-out cross-validation strategy was employed to enhance generalizability across subjects. The results indicated that several classifiers achieve accuracy and area under the curve (AUC) values exceeding 0.95, with random forest and support vector machine-based models demonstrating near-perfect class separability, with an AUC of 0.98. These findings highlighted the effectiveness of using minimal set of biomechanically relevant gyroscope features for gait classification in real-world healthcare applications. The proposed pipeline is computationally efficient, making it well suited for implementing in wearable and remote monitoring systems.

## 1. Introduction

Gait analysis has been defined as the study of human locomotion, offering insights into motor control, functional ability, and the presence of pathological deviations [1,2]. Although observational assessments are common in clinical practice, without dedicated instrumentation they often lack the precision required to detect and quantify subtle gait asymmetries. Traditional gait analysis systems, mostly found in motion analysis laboratories, make use of advanced tools such as optical motion capture, surface electromyography, and force platforms to capture high-resolution biomechanical data [3]. Despite their impressive accuracy, these systems have a set of drawbacks, such as high financial costs, significant space requirements, and complex operational demands, with investments frequently reaching several million dollars [4].

The economic and logistical barrier becomes particularly problematic in the context of stroke rehabilitation, where timely, repeated assessments of motor asymmetry are vital. It is very common for stroke patients to experience hemi-paresis, a medical condition that often leads to asymmetrical gait, identified by unequal stance or swing phases between limbs [5]. The process of detecting and quantifying such asymmetries in a reliable and scalable manner remains a major challenge in both inpatient and outpatient care settings. Although healthcare professionals can perform structural gait assessments during scheduled visits, continuous, high-frequency monitoring throughout a patient’s daily life is not feasible without technological assistance. Early and continuous identification of gait deviations is important for developing personalized rehabilitation programs, tracking recovery progress, and preventing secondary complications such as joint degeneration, falls or further injuries [2]. Recent research has explored the integration of biomechanical modeling and control strategies within assistive and rehabilitation robotics systems, including torque estimation, tracking ability and energy-efficient locomotion support [6,7]. While these approaches address different levels of the rehabilitation pipeline, they highlight the importance of accurate and reliable gait characterization as a fundamental component of adaptive intervention systems.

In the last decade, advances in the Internet of Things (IoT) and embedded computing have increased interest in using wearable sensors—particularly inertial measurement units (IMUs)—to provide low-cost and scalable alternatives to laboratory-based gait analysis systems [8,9,10]. Sensors, including accelerometers and gyroscopes, provide three-dimensional monitoring in everyday environments, creating an ideal case for remote monitoring and rehabilitation [11,12]. Many studies have focused on gait event detection, such as identifying heel strikes or toe-offs and using IMUs. More specifically, Voisard et al. [13] developed automatic gait event detection using both IMU data and an instrumented mat, creating an algorithm capable of operating accurately across healthy individuals and patients with moderate to severe impairments, including those recovering from stroke. Larsen et al. [14] extended this line of enquiry by introducing a neural network-based approach optimized for smartphone-embedded IMUs, achieving high detection accuracy in real-world conditions. These sensors combined with machine learning techniques produce IMU data that can be processed and extract relevant temporal and kinematic features, enabling the classification of abnormal gait signals with high accuracy [15,16].

Studies thus far have used both machine and deep learning models such as support vector machines (SVMs), random forests (RFs), and convolutional neural networks (CNNs) to classify human movement patterns via IMU features [17,18]. The models have shown promising results in general human activity recognition and gait classification tasks. Furthermore, Patterson et al. [5] quantified temporal gait asymmetries among community-ambulating stroke survivors, whereas Zhao et al. [19] presented a dual foot-mounted IMU system for rehabilitation assessment in patients with cerebral thrombosis. Their system used an inequality-constrained ZUPT-aided inertial navigation approach to estimate gait parameters. However, they often rely on extensive datasets, high computational resources, or complicated feature extraction pipelines that limit their applicability in real-time or resource-constrained environments. Additionally, there is a notable lack of lightweight and interpretable methods specifically designed to detect clinically meaningful gait asymmetries, especially in populations recovering from neurological events such as stroke. To date, studies have been designed around generic activity datasets or healthy individuals, overlooking the unique gait deviations, intersubject variability, and clinical needs associated with poststroke rehabilitation [5,20,21].

To address the need for efficient and interpretable gait classification, this work proposes a minimal-feature, machine learning-based pipeline that distinguishes between normal and abnormal gait patterns via statistical extrema from bilateral gyroscope signals. With respect to computational efficiency and transparency, the proposed approach is well suited for both embedded deployment in wearable rehabilitation systems and web-based applications for healthcare professionals. To ensure generalizability across individuals—something which is particularly important for heterogeneous stroke populations—we employed a leave-one-out cross-validation (LOOCV) strategy. The data processing, feature engineering, model design, and evaluation are all original contributions that build upon gyroscope data from a prior study, with the ultimate goal of enhancing the clinical utility of the dataset.

## 2. Materials and Methods

**Figure 1:**
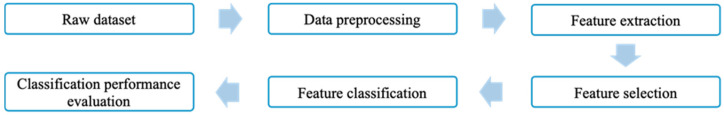
Methodology flowchart of this study.

This study was based on data previously collected and reported by Sanghan et al. [22]; their work involved a comprehensive investigation of the three-dimensional shank kinematics in stroke patients compared with healthy controls. The present analysis was performed under an ethical exemption approved by the Human Research Ethics Unit (HREU) of the Faculty of Medicine, Prince of Songkla University, and in accordance with the ethical principles of the Declaration of Helsinki (REC.68-342-38-2). This study’s methodological workflow is illustrated in Figure 1.

### 2.1. Dataset

**Figure 2:**
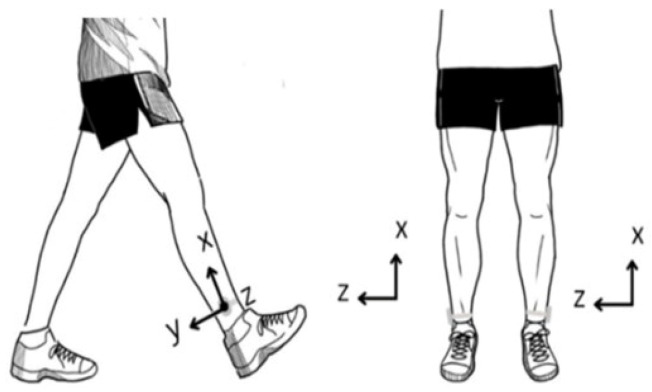
Schematic of IMU sensors’ placement adapted from [22], showing the bilateral attachment of the sensors to the shanks.

In this study, the dataset comprised a total of thirty-two participants, including sixteen stroke patients and sixteen age-matched healthy individuals. Inclusion and exclusion criteria, along with the approval procedure were comprehensively reported in the original study [22]. As described in the previous investigation, all participants were instructed to walk at their self-selected comfortable speed along a flat 10 m indoor walkway while wearing the wireless IMU sensors (MBIENTLAB Inc., San Jose, CA, USA) bilaterally and tightly attached to their shanks, as shown in Figure 2. During walking trails, tri-axial gyroscope data were recorded at a sampling frequency of 100 Hz and transmitted to a laptop via a dedicated smartphone application. The IMU sensors coordinate system was defined such that the *x*-axis corresponds to vertical (up-down), the *y*-axis to anteroposterior (backward-forward), and *z*-axis to mediolateral (left-right) motions. The IMU sensors placed on both shanks were synchronized during data acquisition using a common recording system, ensuring precise temporal alignment between left and right limb signals. Such synchronization is critical for accurately computing temporal gait features, including stance, swing, and stride intervals.

### 2.2. Feature Extraction

**Figure 3:**
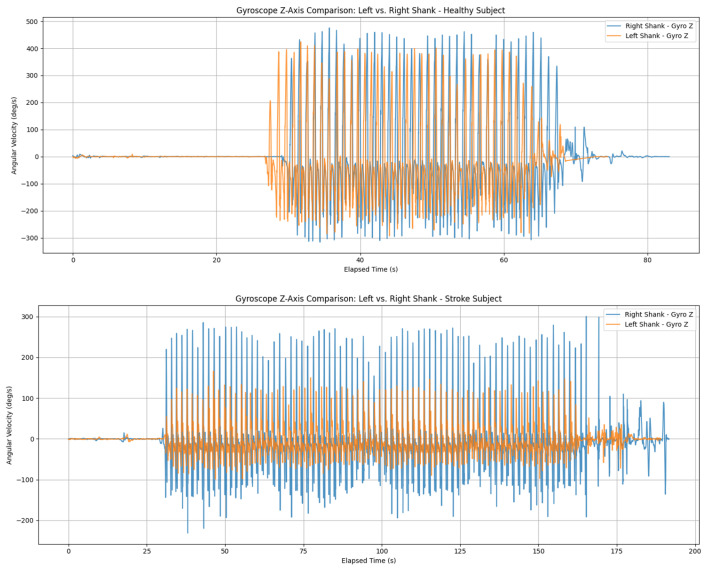
Examples of raw gyroscope signal from healthy subject (**top**) and left-affected side stroke patient (**bottom**).

For features extraction, gyroscope signals acquired from IMU sensors mounted bilaterally on the shanks were analyzed, with particular emphasis on the *z*-axis angular velocity. Figure 3 shows examples of raw gyroscope data from both healthy subjects and stroke patients. This axis is closely associated with the gait cycle, as it reflects a lower-limb flexion-extension motion that is highly indicative of gait abnormalities [22].

The IMU placement on the lateral shank and the use of *z*-axis angular velocity for gait phase identification are consistent with established methods that have demonstrated high accuracy in gait cycle segmentation and spatiotemporal parameter estimation [23,24]. Shank-derived data were analyzed, as signals from other lower- or upper-body segments did not contribute additional discriminative information for the classification task. This methodological decision is supported by Hwang et al. [25], who reported superior gait classification performance using shank-mounted IMUs in joint impairment detection.

To remove high-frequency noise while preserving gait-relevant signal components, all signals were preprocessed prior to feature extraction using a second-order low-pass Butterworth filter with a cutoff frequency of 10 Hz.

To achieve a comprehensive representation of gait dynamics, an initial set of features was extracted across four categories:

- Statistical signal features: mean, standard deviation, minimum, maximum, root mean square (RMS), peak-to-peak amplitude, mean absolute deviation (MAD), skewness, and kurtosis.
- Temporal gait features: stance time, swing time, and stride duration for each limb.
- Motion-based features: limb-specific motion scores derived from angular velocity magnitude, inter-limb motion ratios and overall motion intensity.
- Asymmetry features: normalized differences between left and right limb metrics, including stance, swing, and motion-based asymmetry indices.

Feature extraction was performed independently for each participant, with all features subsequently aggregated at the subject level to form a fixed-length representation suitable for classification.

### 2.3. Feature Selection

Feature selection was performed to identify those most relevant for the classification task. Given the relatively small sample size (*n* = 32) and the high dimensionality of the initial feature space, feature selection was used to identify the most informative and robust predictors. Point-biserial correlation analysis was initially used to assess the relationship between each feature and the binary class label (healthy vs. stroke), providing discriminative strength-based feature ranking. Subsequently, recursive feature elimination (RFE) with a random forest estimator was applied to iteratively remove fewer informative features based on model-derived importance scores. However, due to the limited dataset, both correlation- and model-based feature selection methods exhibited variability in the selected features, indicating potential instability and increased overfitting risk.

To address this, the final feature set was refined using an additional constraint based on biomechanical relevance and robustness. The selected features were as follows:

- Minimum *z*-axis angular velocity (left shank);
- Maximum *z*-axis angular velocity (left shank);
- Minimum *z*-axis angular velocity (right shank);
- Maximum *z*-axis angular velocity (right shank).

These features were selected based on both their empirical performance and biomechanical relevance. More specifically, the minimum and maximum values of the *z*-axis angular velocity were consistently identified as highly discriminative across feature selection methods, while also providing a direct representation of lower-limb rotational dynamics during gait. The *z*-axis angular velocity of the shank has been widely used in prior studies for gait phase identification and spatiotemporal parameter estimation, as it reflects the cyclical flexion–extension behavior of the lower limbs during walking [23,24]. In this context, peak angular velocities correspond to key gait events and limb excursion magnitude, whereas minimum values capture transitional phases associated with deceleration and limb reversal. In post-stroke populations, these extremal characteristics are often altered due to impaired motor control and gait asymmetry, which have been shown to significantly affect spatiotemporal gait parameters [5,20,21]. Therefore, the selected features provide a compact, interpretable, and biomechanically grounded representation that is well suited for distinguishing between healthy and pathological gait patterns, while maintaining computational efficiency for real-time and embedded applications.

While additional statistical features such as standard deviation, RMS, and median absolute deviation were initially considered, they exhibited redundancy and higher variability across subjects in preliminary analysis. In contrast, extremal features (minimum and maximum angular velocity) demonstrated more stable behavior and consistent discriminative capability, making them more robust for subject-level classification.

### 2.4. Classification Models and Evaluation Strategy

Although the total sample comprised 32 participants—which is relatively substantial for a clinical study—the dataset remains limited in size. This constraint, along with the modest dimensionality of the feature space, motivated the use of classical machine learning approached rather than deep learning models. Deep learning models generally require large volumes of training data to achieve robust generalization and are more susceptible to overfitting in small-sample settings. In contrast, conventional machine learning algorithms are well suited to structured datasets of limited size and offer robust performance with greater model interpretability.

Several classifiers were evaluated, including logistic regression, random forest, support vector machines with linear, radial basis function (RBF), polynomial kernels, extreme gradient boosting (XGBoost), and k-nearest neighbors (KNN). These models were chosen to represent a range of learning paradigms, including linear, nonlinear, and ensemble, and instance-based approaches, enabling a comprehensive comparison of classification performance.

To ensure robust evaluation under limited data conditions, leave-one-out cross-validation (LOOCV) was employed. In this approach, each subject is iteratively used as a test sample, while the remaining subjects form the training set. This process is repeated for all participants, ensuring that every sample contributes to both training and evaluation. LOOCV is particularly well suited for small datasets, as it maximizes the use of available training data, provides an approximately unbiased estimate of generalization performance at the subject level, and reduces the instability associated with random data partitioning. Model performance was assessed using standard classification metrics, including accuracy, precision, recall, F1-score, and the area under the receiver operating characteristic curve (AUC).

All models were evaluated using fixed hyperparameters settings selected to balance bias–variance trade-offs in small-sample datasets; the complete parameter settings are reported in Table 1.

## 3. Results

### 3.1. Model Accuracy Metric

Table 2 shows the classification performance of each model in terms of accuracy, precision, recall, F1-score and AUC, using the selected features, including the minimum and maximum *z*-axis angular velocity values from the left and right legs. The evaluated models include logistic regression, random forest, support vector machines with linear, polynomial, RBF kernels, XGBoost, and KNN.

The highest F1-score (0.94) was achieved by the random forest model, followed by the logistic regression and the XGBoost models, which attained 0.90 and 0.91, respectively. The SVM models with linear and RBF kernels yielded moderate performance (F1-score = 0.86) while that with the polynomial kernel demonstrated substantially reduced performance, with an F1-score of 0.72. The KNN model exhibited an F1-score of 0.77, which was slightly higher than that of SVM with polynomial.

An important consideration when evaluating these models is the balance between precision and recall. Several classifiers, including the SVM variants, logistic regression and KNN models, demonstrated very high precision (1.00), indicating highly reliable abnormal gait prediction. However, the SVM models exhibited relatively low recall scores, ranging from 0.56 to 0.75, suggesting that a significant proportion of actual abnormal cases was not detected.

In the context of gait classification for stroke rehabilitation, failing to identify true abnormal gait patterns may be more detrimental than the generation of false-positives. High recall is thus essential for enabling early identification and timely intervention. In contrast, models such as random forest and XGBoost achieved both high precision and high recall, successfully identifying the majority of abnormal gaits while maintaining a low false-positive rate. This performance profile makes these models particularly suitable for deployment in clinical screening applications, where sensitivity is closely linked to patient safety.

### 3.2. Receiver Operating Characteristic (ROC) Curve Analysis

**Figure 4:**
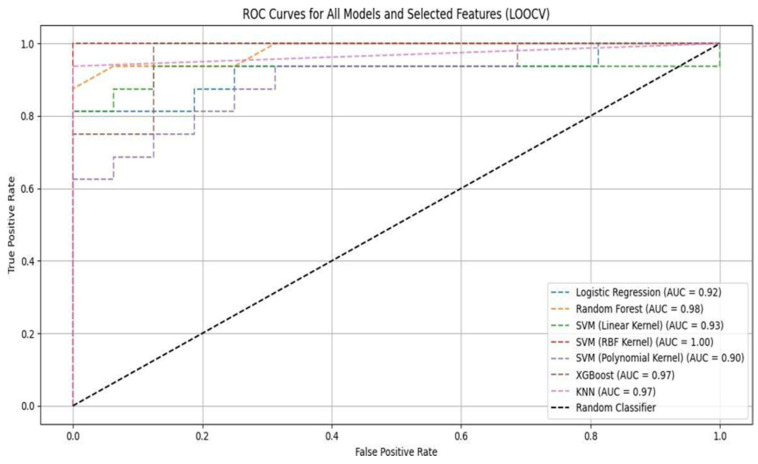
ROC curves for all classifiers using LOOCV.

The ROC curves presented in Figure 4 provide additional insight into each model’s differential ability to distinguish between classes. Notably, the SVM with the RBF kernel achieved a perfect area under the curve (AUC = 1.00), indicating excellent overall separability between normal and abnormal gait classes across all decision thresholds. However, this strong performance was not fully reflected in its recall and F1-score, which remained moderate. AUC reflects threshold-independent ranking performance, whereas recall depends on a specific decision threshold. Therefore, the observed discrepancy arises from threshold selection rather than model performance. In real-world deployment, adaptive thresholding or cost-sensitive decision strategies could be employed to prioritize recall, particularly in screening scenarios where false negatives carry higher clinical risk.

Random forest and XGBoost achieved excellent AUC values of 0.98 and 0.97, respectively, indicating a strong balance between sensitivity and specificity. Notably, despite its linear formulation, logistic regression attained a robust AUC of 0.92, showing that the selected feature set is largely linearly separable. In contrast, although the SVM with a polynomial kernel model exhibited a relatively high AUC of 0.90, its lower F1-score and higher misclassification rate suggest a potential overfitting or misalignment between model complexity and the limited dataset size. While ensemble-based models such as random forest and XGBoost consistently performed well across both the F1-score and AUC, other models (e.g., SVM with the RBF kernel) demonstrated discrepancies between AUC and recall. The ensemble models’ consistent performance across multiple evaluation criteria indicates greater robustness.

### 3.3. Confusion Matrix Evaluation

**Figure 5:**
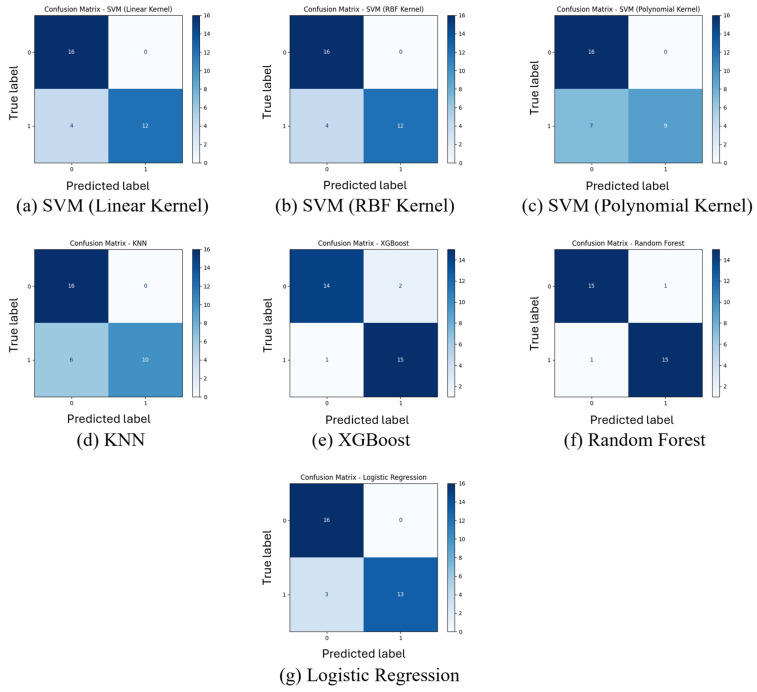
Confusion matrices for all classification models. Each matrix shows the number of true positive, true negative, false positive, and false negative predictions. Class label 0 corresponds to healthy gait, while class label 1 represents post-stroke abnormal gait.

Figure 5 presents the confusion matrices for each classifier. Overall, most models exhibited strong diagonal dominance, indicating effective discrimination between classes. Notably, the logistic regression, random forest and XGBoost models each misclassified mostly one instance, resulting in low false positive and negative rates. The SVM variants demonstrated moderate performance, with the RBF and linear kernels achieving better classification than the polynomial kernel. These models showed a tendency to misclassify abnormal gaits as normal, resulting in a higher false-negative rate; this is a crucial consideration in clinical settings where missed pathological gaits are more consequential than their over-detection. The KNN model exhibited the highest number of misclassifications, with a notable number of false positives and negatives, indicating limited robustness for this classification task. Overall, these confusion matrices reinforce the findings and emphasize the consistency and clinical usability of ensemble-based classifiers such as random forest and XGBoost for reliable gait classification via minimal yet meaningful features.

## 4. Discussion

A comparative analysis of the machine learning classifiers provides insight not only into performance differences, but also into how model behavior interacts with the low-dimensional, subject-level feature space used in this study. The *z*-axis angular velocity reflects the rotational motion of the shank in the sagittal plane, which is directly associated with forward leg swing and push-off dynamics. Because the proposed framework relies on aggregated extremal gyroscopic features rather than high-frequency temporal representations, model performance is primarily governed by robustness to inter-subject variability, sensitivity to noise, and the ability to generalize from limited samples. While explicit decision thresholds vary across models, classification is primarily driven by differences in extremal angular velocity values between healthy and post-stroke gait patterns. In our previous study, abnormal gait was associated with reduced peak angular velocities and increased asymmetry between limbs [22]. These characteristics form the implicit decision boundaries learned by the models and provide clinically interpretable gait impairment indicators.

Among the evaluated models, random forest, XGBoost, and logistic regression consistently demonstrated superior performance across multiple evaluation metrics. The random forest model achieved the highest F1-score (0.94) and exhibited near-perfect classification performance, with only a single misclassified instance observed in the confusion matrix. This performance can be attributed to the ensemble learning framework, which aggregates multiple decision trees to mitigate overfitting and effectively capture complex, nonlinear patterns within the data. Similarly, XGBoost demonstrated excellent generalizability, with a high AUC of 0.96 and minimal misclassification, owing to its gradient-boosted tree approach, which optimizes classification boundaries on the basis of prior errors. Logistic regression, while a linear model, also performed competitively (F1-score of 0.90), likely reflecting the strong discriminative characteristics of the selected features and the low-dimensional nature of the dataset. In contrast, the SVM models, particularly the polynomial kernel variant, exhibited comparatively lower performance. While the SVM models with linear and RBF kernels achieved reasonable AUC values (0.92 and 1.00, respectively), their F1-scores remained moderate (0.86) and accompanied by a higher false-negative rate. This tendency to misclassify abnormal gait instances as normal is a critical limitation in clinical contexts, where sensitivity to pathological gait patterns is essential. The observed performance gap can be attributed to the sensitivity of SVM models to feature scaling, kernel parameter tuning, and decision boundary rigidity in smaller datasets. Finally, the KNN model also demonstrated a lower overall performance. Its reliance on instance-based learning likely increased its susceptibility to local noise and feature overlap between classes, as reflected by the elevated number of misclassifications. Compared with prior studies, our study achieved comparable or improved classification performance while utilizing only four gyroscope-derived features (maximum and minimum angular velocities from both shanks) extracted from a single IMU per leg. For instance, Trojaniello et al. [11] used seven kinematic channels to achieve classification accuracy exceeding 90%, while Pohl et al. [26] employed both wrist and ankle IMUs and reported SVM performance of up to 93%. Other studies, including those proposed by Zhao et al. [19] and Weidong and Zhenwei [27], relied on complex pipeline architectures involving ZUPT-aided navigation or fuzzy logic combined with empirical mode decomposition; although effective, these approaches are computationally intensive and not easily deployable in real-world clinical settings.

In contrast, the proposed feature set is intentionally minimal, interpretable, and biomechanically meaningful. By focusing on extremal angular velocity values that directly relate to shank rotation during the gait cycle, the framework preserves discriminative power while reducing sensitivity to sensor noise, feature redundancy, and overfitting. Compared to deep learning approaches, it operates on a significantly reduced feature set and requires minimal computational resources for both training and inference. While deep models often demand substantial training time and hardware acceleration, the models used in this study can be trained and executed on standard computing devices with negligible latency. Although exact timing benchmarks were not the focus of this study, the simplicity and efficiency of the proposed framework support its suitability for real-time deployment in embedded and wearable systems operating under computational, energy, and bandwidth constraints.

From an application perspective, the proposed framework is computationally lightweight due to the use of a minimal feature set and classical machine learning models. This enables both training and inference on low-resource devices, supporting potential deployment in wearable systems for the screening-level detection of gait abnormalities and remote monitoring.

In addition, while deep learning models such as LSTM have shown promises for achieving approximately 95% performance in stroke gait phase detection, their reliance on large datasets and computational resources limits their feasibility for real-time, rehabilitation applications [28]. Our results show better performance in cases where the feature set is thoughtfully selected based on domain knowledge; classical models can offer excellent generalizability even with small sample sizes. However, the relatively small dataset size (*n* = 32) could introduce inherent overfitting risk, particularly for more flexible models such as ensemble methods. This risk was mitigated through the use of low-dimensional feature representations, subject-level cross-validation, and consistent performance across multiple evaluation metrics. Additionally, the strong performance of simpler models such as logistic regression suggested that the classification task was well-structured and not solely driven by model complexity.

It is important to acknowledge the several limitations of this study. Firstly, the dataset size is relatively small, which may limit the generalizability of the findings and increase sensitivity to inter-subject variability. Furthermore, the absence of external validation against an independent dataset limits our ability to fully assess model generalizability across different populations and acquisition conditions. This limitation is partly due to the lack of publicly available datasets that provide comparable bilateral shank-mounted IMU measurements in post-stroke populations, which in turn restricts our ability to perform cross-dataset validation. Statistical significance testing between models was also not tested but will be considered in future work to provide more rigorous comparative analysis. Secondly, while beneficial for robustness and simplicity, the use of subject-level aggregated features does not capture intra-subject variability or temporal dynamics within individual gait cycles.

Overall, the results indicate that a minimal feature set—focused on clinically meaningful statistics such as the maximum and minimum angular velocities of the lower limbs—can lead to high predictive performance when paired with appropriate classifiers. The effectiveness of combining simple IMU-derived signals, a computationally efficient pipeline, and classical machine learning models highlights the important role of domain understanding in developing scalable and clinically applicable artificial intelligence solutions for gait analysis.

## 5. Conclusions

This study presents a lightweight and effective machine learning framework for classifying abnormal gait patterns using a minimal set of clinically meaningful features derived from gyroscope data. While the present work focuses on binary classification between normal and abnormal gait, this design choice reflects the intended role of the proposed framework as a screening-level detection tool. Binary classification enables the early identification of pathological gait patterns in resource-constrained, wearable settings, where computational efficiency and robustness are prioritized.

Among the evaluated classifiers, the random forest, XGBoost, and logistic regression models achieved the strongest overall performance in terms of accuracy, F1-score, and AUC, reflecting robust generalization capabilities and low misclassification rates. In contrast, the SVM-based models exhibited lower sensitivity, particularly in detecting abnormal gaits, underscoring the importance of algorithm selection in clinical applications. The findings emphasize how, even with limited features and small datasets, robust classification is achievable through proper feature engineering and algorithm choice. Future work will focus on extending the proposed framework to include a comparative analysis across multiple IMU axes and modalities (e.g., gyroscope and accelerometer) to better evaluate their complementary contributions to gait characterization. Additionally, the framework will be integrated into real-time gait rehabilitation systems, with on-device inference capabilities for wearable platforms. In parallel, multi-class severity classification (e.g., mild, moderate, or severe impairment) will be addressed in a companion study, which extends the present pipeline by incorporating accelerometer-derived features and severity aware modeling.

## Figures and Tables

**Table 1:** Hyperparameter settings for evaluated classification models.

Model	Key Parameters
Logistic Regression	L2 penalty; solver: liblinear; C = 1.0
Random Forest	Number of trees: 100; maximum depth: none; random state: 42
SVM (Linear Kernel)	C = 1.0; linear kernel
SVM (RBF Kernel)	C = 1.0; RBF kernel; gamma = scale
SVM (Polynomial Kernel)	C = 1.0; polynomial kernel; degree = 3
XGBoost	Number of estimators: 100; learning rate: 0.1; maximum depth: 3
KNN	Number of neighbors: 5; distance metric: Euclidean

**Table 2:** Classification performance metrics of the evaluated models using LOOCV.

Model	Accuracy	Precision	Recall	F1-Score
Logistic Regression	0.91	1.00	0.81	0.90
Random Forest	0.94	0.94	0.94	0.94
SVM (Linear Kernel)	0.88	1.00	0.75	0.86
SVM (RBF Kernel)	0.88	1.00	0.75	0.86
SVM (Polynomial Kernel)	0.78	1.00	0.56	0.72
XGBoost	0.91	0.88	0.94	0.91
KNN	0.81	1.00	0.62	0.77

## Data Availability

All data, models, and code generated or used during the study are available from the corresponding author upon reasonable request.
